# Integrative omics analysis identifies biomarkers of septic cardiomyopathy

**DOI:** 10.1371/journal.pone.0310412

**Published:** 2024-11-15

**Authors:** Kexin Cai, Yuqing Luo, Hongyin Chen, Yanfang Dong, Yunyun Su, Chen Lin, Chuanqi Cai, Yikbin Shi, Siming Lin, Guili Lian, Zhihong Lin, Shaodan Feng

**Affiliations:** 1 Department of Emergency, The First Affiliated Hospital of Fujian Medical University, Fujian, Fuzhou, China; 2 Fujian Hypertension Research Institute, The First Affiliated Hospital of Fujian Medical University, Fujian, Fuzhou, China; 3 Department of Emergency, The Third Affiliated People’s Hospital, Fujian University of Traditional Chinese Medicine, Fuzhou, China; 4 Department of Emergency, National Regional Medical Center, Binhai Campus of The First Affiliated Hospital, Fujian Medical University, Fujian, Fuzhou, China; Pacific Northwest National Laboratory, UNITED STATES OF AMERICA

## Abstract

Septic Cardiomyopathy (SCM) is a syndrome of acute cardiac dysfunction in septic patients, unrelated to cardiac ischemia. Multiomics studies including transcriptomics and proteomics have provided new insights into the mechanisms of SCM. In here, a rat model of SCM was established by intraperitoneal injection of lipopolysaccharide (LPS). Biomarkers of SCM were characterized via a multi-omics analysis. The differentially expressed (DE) mRNAs predominantly appeared in pathways linked to the immune response, inflammatory response, and the complement and coagulation cascades, while DE proteins were mainly enriched in pathways associated with the complement and coagulation cascades. On this basis, the integrated analysis was performed between transcriptome and proteome. The potential biomarkers were further verified by RT-qPCR and WB. The current proteotranscriptomic research has furnished a valuable dataset and fresh perspectives that will enhance our comprehension of the development of SCM. This, in turn, is expected to expedite the formulation of novel approaches for the prevention and management of SCM in patients.

## Introduction

Sepsis is characterized as a critical organ malfunction resulting from an uncontrolled host reaction to infection [1,2]. Epidemiological studies reported that between 28.3% and 41% of patients with sepsis die of multiple organ failure after hospital admission [3]. Due to its high morbidity and mortality rates, sepsis is considered among the top ten leading causes of death worldwide [4], especially lethal among critically ill patients, with 50 million cases and 11 million deaths per year [5].

In cases of sepsis, the fate of each organ is linked to the others such that the failure of one organ typically triggers dysfunction or failure of the others. This interdependence is particularly evident during cardiovascular failure, emphasizing the necessity of understanding cardiac dysfunction in sepsis [6]. Yet, the complexity of the cardiovascular system, the multitude of cardiac dysfunction assessment methods, and the variability of the heart’s pre-septic state make elucidation of causality challenging. The clinical diagnosis of septic cardiomyopathy (SCM) still heavily relies on echocardiography and markers of myocardial injury, which currently do not allow for early intervention [7]. Therefore, the discovery of new biomarkers for early diagnosis and intervention may help to reduce mortality in patients with SCM.

Discovering new pathogenic pathways, as well as treatment targets represents a valuable application of high-throughput genomic techniques like transcriptomics and proteomics [8]. Transcriptomics, a method that quantitatively and qualitatively analyzes mRNA levels across the genome, has been employed in examining a range of illnesses such as diabetes or several cardiovascular disorders [9–11]. Proteomics is a promising approach to directly study expressed proteins and their functions in a cellular context [12,13]. Proteomics-based techniques have been instrumental in understanding the pathogenic mechanisms and interpreting functional pathways underlying a variety of diseases, as well as in the detection of diagnostic markers [14]. Integrating transcriptomics and proteomics datasets has the potential to enhance our comprehension of the processes involved in the development and progression of SCM. Consequently, this integration can lead to the development of more targeted treatment strategies for SCM.

Several studies have demonstrated that the injection of lipopolysaccharide (LPS) through the peritoneal cavity effectively triggers septic myocardial injury in several animal models [15–17]. We employed this approach, along with a multi-omics analysis that incorporates transcriptomics and proteomics data, to better understand the pathogenesis of SCM and to offer theoretical backing and insight for the treatment of SCM.

## Materials and methods

### Experiment design and sample collection

This study was carried out in strict accordance with the recommendations in the Guide for the Care and Use of Laboratory Animals of the National Institutes of Health. This proposal was assented to the Laboratory Animal Welfare and Ethics Committee of Fujian Medical University, Fuzhou, Fujian, China (Protocol Number: MRCTA, ECFAHB of FMU [2022] 244). The Sprague-Dawley rats (male, 200–250 g for each) were bought from the company (Beijing Huifang Biotechnology, China). They have free access to food and water and were raised in a pathogen-free facility (22 ± 2°C, 55 ± 10% humidity, 12-hour light/dark cycle). One week later, they were stochastically separated into the LPS group and the control (Ctrl) group, with 6 in each group. The LPS rats were injected 10 mg/kg of LPS intraperitoneally, and the Ctrl rats were injected 0.9% saline in the same way and same volume. Rats from the same group were raised under the same condition, with six rats per cage. 24 hours after injection, all animals were euthanized by cervical dislocation under sodium pentobarbital anesthesia, and all efforts were made to minimize suffering. The blood and hearts of rats were collected for subsequent analyses. The rat SCM model was established according to our previously reported procedures [18].

Myocardial tissue from each rat was separately fixed in 4% paraformaldehyde, followed by gradual dehydration in an ethanol solution, paraffin embedding, 4-μm sections preparation, hematoxylin-eosin (HE) staining, and photography under a light microscope (Nikon, Japan). A portion of the remaining myocardial tissue was preserved on TRIzol reagent (Invitrogen, China) for RNA extraction, the rest was immediately snap-frozen and stored at -80°C for subsequent protein extraction.

### RNA-Seq library preparation, sequencing, and transcriptomic analysis

Five samples were randomly chosen from each group. Total RNA was extracted from myocardial tissue following a previously established protocol [19]. 2 μg of total RNA was utilized to construct RNA-seq library using KC^TM^ Stranded mRNA Library Prep Kit for Illumina^®^ according to the manual. PCR products ranging between 200 and 500 bps were collected and quantified. The raw data obtained from the sequencer was saved in FASTQ format. Raw reads were quality-filtered using fastp (version 0.23.0) software. Good quality reads were mapped to the reference genome of the domestic rat (Rattus norvegicus), obtained from the National Center for Biotechnology Information (NCBI) database (Rattus_norvegicus.Rnor_6.0, NCBI RefSeq assembly GCF_000001895.5) employing the STRA software (version 2.5.3a) with standard settings. The featureCounts software (Subread-1.5.1) in the R software environment (version 3.12.1) was used to tally the reads that aligned with the exon regions of each gene. Gene expression levels were standardized and reported as Reads Per Kilobase per Million mapped reads (RPKM). Differential gene expression analysis between the two experimental groups (LPS and Ctrl) was performed using the DESeq2 package in R (version 3.12.1). Genes with a fold change ≥ 2.0 and a *p-*value < 0.05 by t-test were used to identify significantly differentially expressed genes (DEGs).

### Proteomic analysis by label-free quantification

Five samples were randomly selected from each group for Label-free protein quantification. Briefly, the myocardial tissues were mixed with Radioimmunoprecipitation assay lysis buffer, protease inhibitors, and phenylmethylsulfonyl fluoride and left to thaw on ice. Then, the myocardial tissue lysates were centrifuged at 14,000 x g and 4°C, and the supernatants were collected. Following this, the supernatants underwent acetone protein precipitation, subsequent protein redissolution, reduction, alkylation, enzymatic hydrolysis, sodium deoxycholate removal, and desalting were carried out. Peptides were then isolated utilizing an EASY-nLC 1200 nano-UPLC liquid phase system and analyzed using a Q-Exactive mass spectrometer. The raw data obtained were processed via MaxQuant software. The Rattus norvegicus proteome data set used in the proteomic analysis was obtained from the UniProt database (uniprot-proteome-rat-10116-UP000002494-2023.4). Proteins with a fold change ≥ 1.5, *p-*value < 0.05 by t-test, and unique peptide ≥ 2 were used to detect differentially expressed proteins (DEPs).

### Pathways analysis

Gene Ontology (GO) and Kyoto Encyclopedia of Genes and Genomes (KEGG) pathways analyses were used to determine the roles of the DEGs and DEPs. Particularly, GO analysis was employed to classify DEGs and DEPs based on the biological processes (BP), cellular components (CC), and molecular functions (MF) they were involved with.

### Real-time quantitative polymerase chain reaction (RT-qPCR) assay

Double-strand cDNA was obtained using the HiScript II Q RT SuperMix for qPCR Kit (Vazyme, China) following the manual. RT-qPCR was carried out using a ChamQ SYBR qPCR Master Mix Kit (Vazyme, China) via a Light Cycler 96 system (Roche, Switzerland). GAPDH was used as house-keeping gene. The primers used in this study are shown in S1 Table.

### Western blot (WB) analysis

Total proteins were extracted from rat myocardial tissue using RIPA lysis buffer (Beyotime, Shanghai, China) and separated on sodium dodecyl-sulfate polyacrylamide gel electrophoresis gel before being transferred to methanol-activated polyvinylidene fluoride membranes (Merck Microporous, Germany). Following a 2-hour blocking period with 5% skimmed milk, the membranes were incubated overnight at 4°C with primary antibodies against GAPDH (1:5000, Proteintech, China); Hp (1:2000, Abcam, UK); FGA (1:2000, Abcam, UK); A2m (1:2000, Abcam, UK); Orm1 (1:1000, Proteintech, China); Hspb1 (1:1000, Proteintech, China); C2 (1:1000, Cloud-Clone Corp., TX, USA). The following day, following three rinses with Tris-buffered saline containing 0.1% Tween-20 buffer, the membranes underwent incubation with a peroxidase-conjugated secondary antibody (SA00001-1 and SA00001-2, Proteintech, China) for a duration of 1 hour at 37°C. Subsequently, the protein bands were visualized using an Enhanced Chemiluminescence Detection Kit (Proteintech, Wuhan, China), captured utilizing iBright 1500 (Thermo Fisher Scientific, Waltham, USA), and quantified with Image J software.

### Statistical analysis

Statistical analysis was performed with GraphPad Prism 9 (GraphPad Software Inc., San Diego, CA). The values were presented as the mean ± standard error of the mean (SEM), and the Student’s *t*-test was used for comparison between groups. Results are statistically significant with *p-*value < 0.05.

## Results

### Histopathological evaluation of cardiac tissue

**Fig 1:**
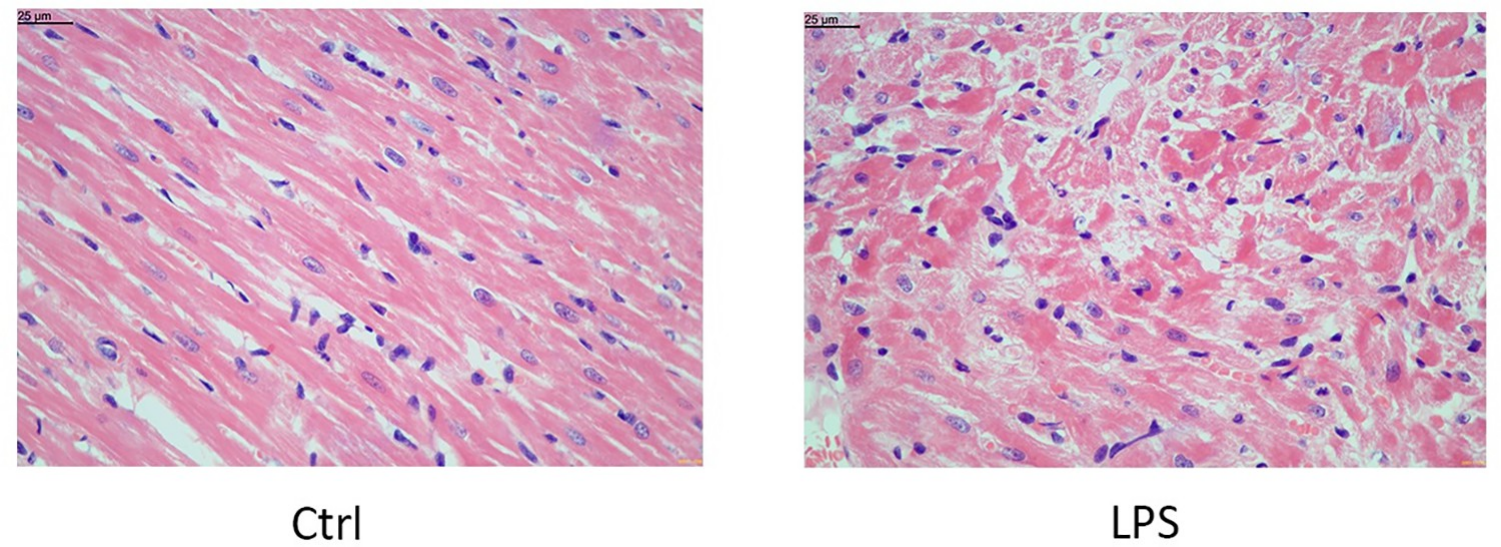
Histological changes of the myocardial tissue assessed by HE staining. Magnification, X400.

Cardiac tissues were normal in the Ctrl group. In contrast, swelling and degeneration of cardiomyocytes were observed in all rats from the LPS-injected group, indicating the successful replication of SCM in the rats of our study (Fig 1). The LPS-induced changes found in the cardiac tissues were detailed in our previous study [18].

### Transcriptomic analysis of the myocardium of rats suffering SCM

An average of 53,156,900 ± 4,268,830 raw reads and 43,935,142 ± 3,763,568 quality-filtered reads were obtained on average per sample. There were no significant differences between the Ctrl group and the LPS group in the number of quality-filtered reads (*p-*value > 0.05). The average GC ratio (GC%) of raw reads was 50.35 ± 0.28%, and the average number of raw reads that passed the Q20 and Q30 quality thresholds were 97.05 ± 0.16% and 90.83 ± 0.44% respectively, evidencing the good quality and reliability of the data. Similarly, there were no significant differences in these three indicators between the two treatment groups (*p-*value > 0.05), and the reads quality was further improved after data quality filtering, showing and effective rate of 82.62 ± 0.99% (Table 1). In addition, principal component analysis (PCA) is shown in S1 Fig.

**Table 1:** Quality control of raw RNA-seq data.

Sample	Ctrl	LPS	p-value
**Raw Reads**	51116482±4927072.04	55197318.4±3297419.66	0.1623
**Raw Bases(G)**	7.67±0.74	8.28±0.5	0.1626
**Raw Q20(%)**	97.02±0.22	97.07±0.1	0.711
**Raw Q30(%)**	90.76±0.62	90.9±0.28	0.6674
**Raw GC (%)**	50.45±0.23	50.26±0.34	0.3374
**Clean Reads**	42218865.6±4441691.51	45651418.8±2884177.49	0.1853
**Clean Bases(G)**	6.03±0.62	6.54±0.39	0.1612
**Clean Q20(%)**	98.92±0.06	98.94±0.03	0.6133
**Clean Q30(%)**	95.38±0.25	95.46±0.11	0.5219
**Clean GC (%)**	50.41±0.25	50.24±0.35	0.4019
**Effective Rate (%)**	82.54±1.43	82.69±0.65	0.8381

This table lists several indices of RNA-Seq. The “LPS” and “Ctrl” columns indicate the results for each group.

**Fig 2:**
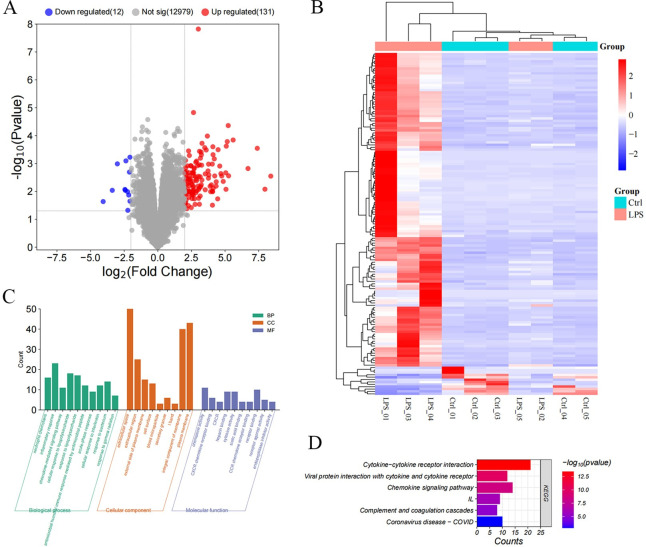
DE mRNAs and enrichment analysis. **A.** Volcano plot of DE mRNAs. **B.** Heatmap of the expression of DE mRNAs. **C.** Bar plot of the GO enrichment analysis, classified into BP, CC, and MF. **D.** Bar plot of the enriched KEGG pathways.

In total, 633 DEGs between the treatment groups were identified when |log_2_ (fold change)| ≥ 1 and *p-*value < 0.05 were applied, and 143 when a more restrictive threshold was used (|log_2_ (fold change)| ≥ 2, *p-*value < 0.05) (S2 Table). Out of these 143 DEGs, 131 genes were found to be significantly up-regulated in the LPS group, whereas 12 genes were significantly down-regulated (Fig 2A and S3 Table). The clustered heatmap (Fig 2B) revealed a distinct separation between the Ctrl and LPS groups based on gene expression, with LPS-injected rats 1, 3, and 4 showing a particularly pronounced contrast with controls. GO annotation and enrichment analysis were performed to discern crucial terms related to the occurrence and progression of SCM (Fig 2C). Additionally, KEGG enrichment analysis was carried out to assess the significant signaling pathways of DEGs. The outcomes of the KEGG enrichment analysis indicated enrichment of the complement and coagulation cascades and cytokine-cytokine receptor interaction (Fig 2D).

### Proteomics analysis of the myocardium of rats suffering SCM

**Fig 3:**
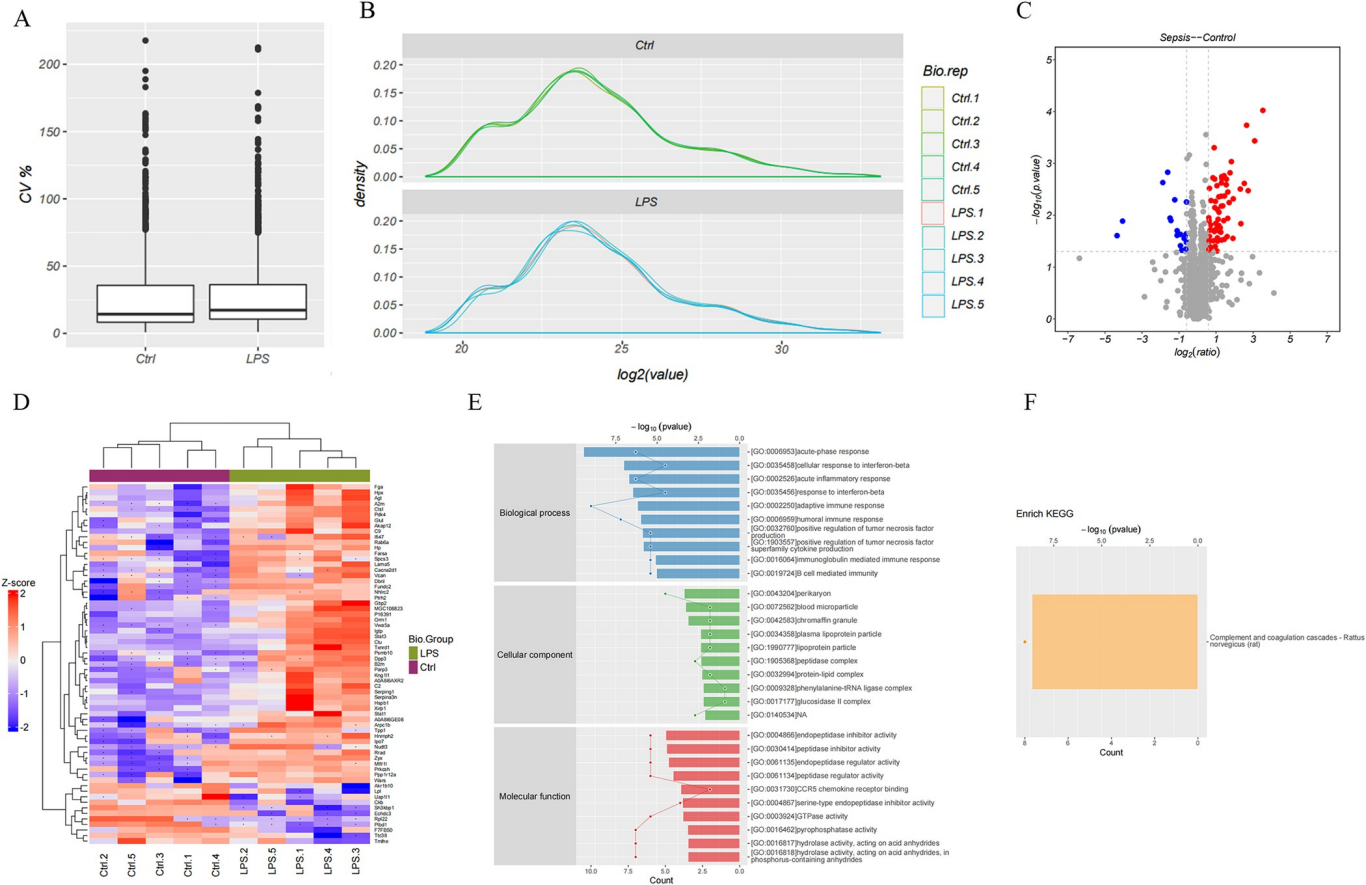
DE proteins and enrichment analysis. **A.** The average CV between the Ctrl and LPS groups. **B.** The standardized display data between the Ctrl and LPS groups. **C.** The volcano plot showed DE proteins. **D.** Heat map of DE proteins. **E.** Bar plot of the GO enrichment analysis, classified into BP, CC, and MF. **F.** Bar plot of the enriched KEGG pathways.

18,344 peptides and 2,114 proteins were obtained at all quality control (QC) (Spectrum, Peptide, and ProteCVn levels). Of these, a total of 1,688 quantifiable proteins were detected (S4 Table). The quantitative coefficient of variation (CV) of the quantifiable proteins was below 25% for both the Ctrl and LPS groups, and the standardized display data indicates good reproducibility (Fig 3A and 3B).

Applying a threshold of fold change ≥ 1.5, p-value < 0.05, and unique peptide ≥ 2, we identified 65 DEPs in SCM tissues (Fig 3C and S5 Table). Among these, 54 were up-regulated, while 11 were down-regulated proteins. The results show protein expression patterns distinguishable between the Ctrl and LPS groups (Fig 3D). Fig 3E illustrates the top ten enriched DEPs across three categories: BP, MF, and CC. It is noteworthy that these enriched gene clusters were primarily related to the immune response and inflammatory processes, which are closely associated with the pathogenesis of SCM. Specifically, DEPs were significantly enriched in the complement and coagulation cascades based on the results obtained in the KEGG pathways analysis (Fig 3F).

### Integration of transcriptome and proteome datasets

**Fig 4:**
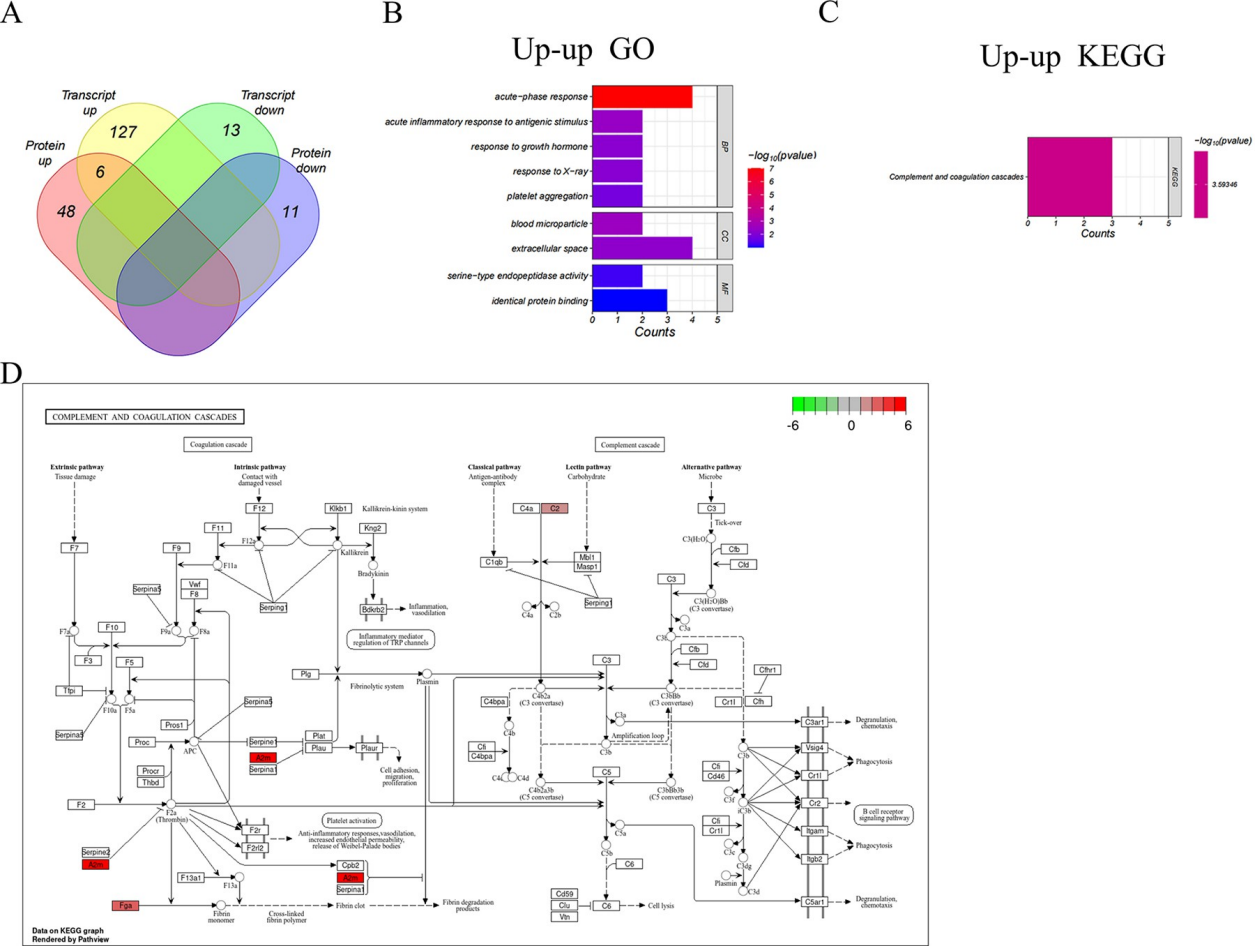
DE genes in both omics and enrichment analysis. **A.** The Venn diagram shows the overlap between DEGs and DEPs, leading to 6 potential biomarkers. **B.** BP, CC, and MF of 6 genes in the up-up group. **C.** KEGG analysis of 6 genes in the up-up group. **D.** Inferred mechanisms of action of the cor-DEPs-DEGs genes with mainly enrichment pathways in the LPS-treated rats. Red frame: Upregulated; Green frame: Downregulated.

To explore the concordance between mRNA and protein levels, we analyzed the two omics data according to the transcript ID converted from the according protein ID. 12 genes were down-regulated at transcript level with no significant protein-level change (down-unchange group), and 125 genes belong to up-unchange group. Six genes were up-regulated at both protein- and transcript-level (up-up group), but no down-down group members (Fig 4A). Although there were only 6 significantly co-expressed differential targets in the transcriptome and proteome (Table 2), the biological processes represented by DEGs and DEPs were remarkably similar. The genes in the up-up group were most enriched in the extracellular space (CC), acute-phase response and inflammatory response (BP), and shared identical protein binding (MF) (Fig 4B). The most significantly enriched KEGG signaling pathways were the complement and coagulation cascades (Fig 4C). These findings validate that processes exhibiting alterations at the transcriptional level are also disrupted at the protein level. A thorough examination of the pathways reveals their interconnectedness and convergence at shared points, resulting in a collective effect (Fig 4D).

**Table 2:** Expression of candidate genes in 6 common DEGs and DEPs.

Gene name	Transcriptomes		Proteomes	
	Log2 FC	p-value	Log2 FC	p-value
**A2m**	5.165923909	0.00016443	5.759950116	0.00242891
**C2**	2.033021155	0.002050027	2.070907106	0.002695498
**Fga**	3.038068356	0.017683011	1.560327231	0.0255391
**Hp**	3.093456723	0.029024009	8.489131926	0.000367299
**Hspb1**	2.89781682	0.009578121	1.937076151	0.019218009
**Orm1**	3.764366578	0.003366333	11.55526047	9.47867E-05

### Identification of potential biomarkers

**Fig 5:**
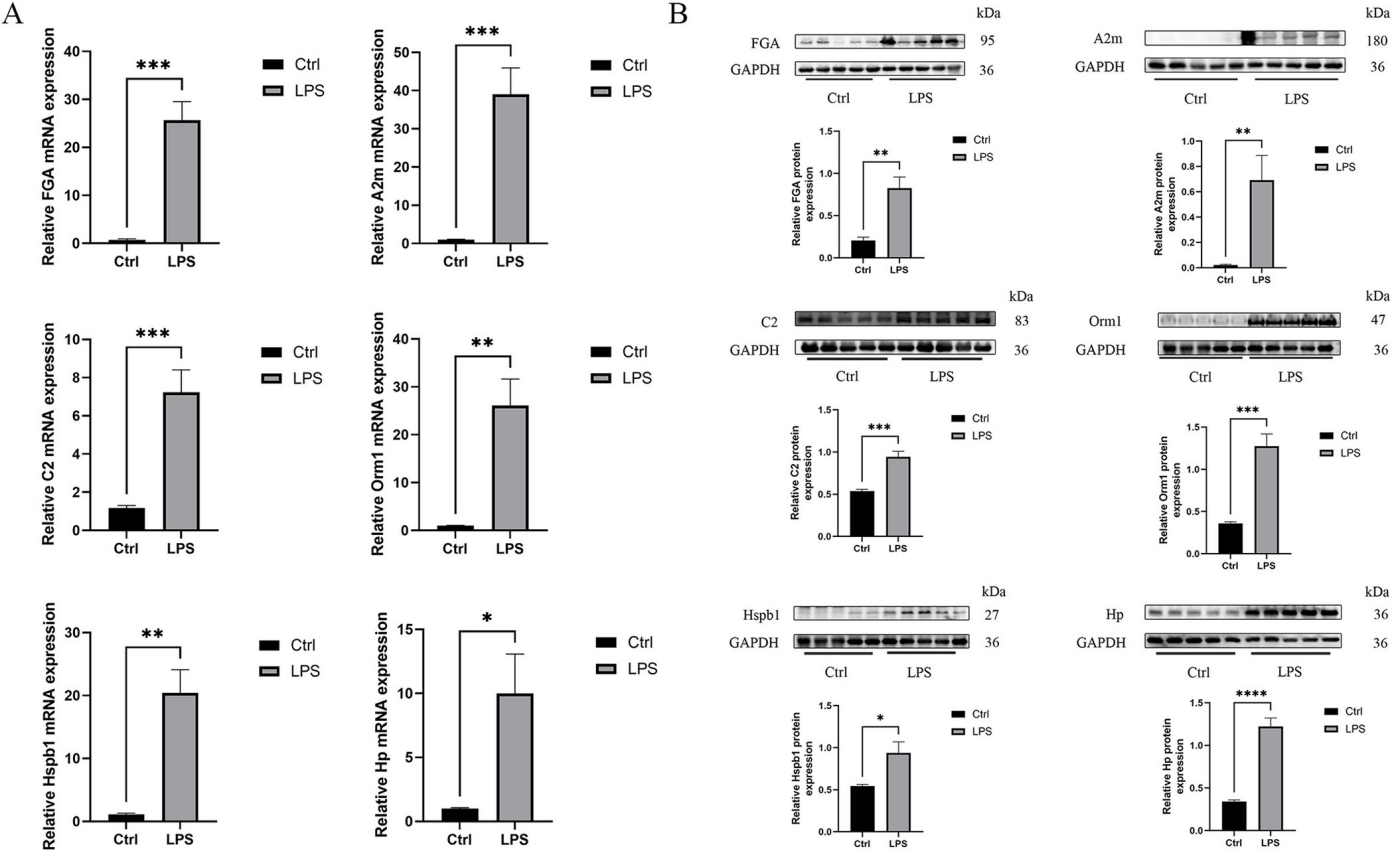
Identification of potential biomarkers. **A.** RNA levels of potential genes in the Ctrl group and the LPS group rats using RT-qPCR (n = 5). **B.** Protein levels of potential genes in the Ctrl group and the LPS group rats using WB (n = 5). Bands were visualized and quantified using Image J. Data are expressed as mean ± SEM (n = 5); ns no significant; * *p* < 0.05, ** *p* < 0.01, *** *p* < 0.001.

The mRNA levels of commonly expressed genes were measured using qPCR, and their protein expression in heart tissues was analyzed using WB. qPCR assay showed that mRNA expression of Fga, A2m, C2, Orm1, Hp and Hspb1 was significantly elevated in the LPS group (Fig 5A). WB assay showed that protein expression of Fga, A2m, C2, Orm1, Hp and Hspb1 was all significantly elevated in LPS-induced rats (Fig 5B). We found that these data exhibited a consistent trend with the bioinformatics analysis. Differences in expression levels (e.g., fold changes) may arise from different assay strategies.

## Discussion

SCM is considered a common but extremely serious complication of sepsis [20]. While the dysregulation of the host’s immune response is linked to the impairment of organ function, the pathophysiological mechanisms underlying sepsis-induced cardiac alterations are intricate and influenced by various factors, the pathogenesis of which remains unclear. This study aimed to systematically investigate the molecular mechanism underlying SCM in rats by analyzing the transcriptomic and proteomic profiles of heart tissue. We characterized the changes and related biological pathways of various genes and proteins in the rat heart.

We utilized RNA-seq and label-free protein quantification to analyze DEGs and DEPs in rat myocardial tissue. Six cor-DEPs-DEGs genes were identified and validated by RT-qPCR and WB in the myocardial tissues of the LPS-treated rats compared to the Ctrl group. Since the 1970s, the pathogenesis of sepsis has often been associated with excessive immune inflammation, the so-called "cytokine storm" [21,22]. This association has notably raised questions about the potential role of bacterial superantigens in driving pathogenic "cytokine storms", although the exact evolutionary function of these superantigens remains uncertain [23,24]. In the up-up group, the enrichment of GO terms such as “acute-phase response” and “acute inflammatory response to antigenic stimulus” was significant. This suggests that the hearts of rats experiencing SCM were in a state of heightened immune activation, and indicates an excessive immunological response. In the early stages of sepsis, although the body initiates both pro-inflammatory and anti-inflammatory mechanisms, the pro-inflammatory response tends to dominate, leading to an intense hyper-inflammatory condition. This state can precipitate multi-organ failure in as little as 1–2 days and may ultimately result in the patient’s death [25–27].

We observed that the majority of rats injected with LPS showed significant enrichment of the complement and coagulation cascade pathways (Fig 4D). Several studies have highlighted the close interplay between the coagulation and complement systems, indicating that uncontrolled activation of these enzyme cascades could severely impair organ function and lead to fatalities in cases of sepsis [8,28–30]. These findings suggest that the coagulation and complement pathways may have crucial implications in the development of SCM.

The identification of potential biomarkers involved in the pathogenesis of SCM is critical for understanding the disease and developing new diagnostic markers. The complement and coagulation cascades are key mediators of innate immunity and blood coagulation respectively. Notably, *A2m*, *C2*, and *Fga* were enriched in complement and coagulation cascade reactions (Fig 4D). *A2M* (Alpha-2-macroglobulin), an extracellular macromolecule, is primarily recognized for inhibiting broad-spectrum proteases [31]. Several clinical studies have found highly abnormal *A2M* in critically ill septic patients [32–34], and a direct correlation between *A2M* and acute-phase protein and alkaline phosphatase levels, providing insight into the pathophysiology of septic myocardial injury [35]. Fibrin can modulate inflammation by altering leukocyte activity, and *Fga* is an important component of fibrinogen that reflects the severity and predicts the prognosis of patients with sepsis and septic shock [36,37]. In addition, *C2* has been reported to activate the classical complement pathway primarily by inhibiting other alternative pathways [38]. In addition, *Orm1* (Alpha-1-acid glycoprotein) is produced by liver and peripheral tissues. Several studies have shown that *Orm1* has a significant protective effect on several models of inflammation [39,40]. *Hp* (Haptoglobin) has been reported to be strongly associated with infectious and non-infectious (e.g. cardiovascular diseases) diseases [41,42]. Both *Hp* and *Orm1* are acute phase proteins. Study of the Orm1-Hp couple may be useful for the diagnosis of endocarditis [43]. *Hspb1* (Heat shock 27kDa protein 1) is a molecular chaperone that possesses the capacity to engage with a wide array of proteins. Recent findings indicate that *Hspb1* controls apoptosis by virtue of its ability to interact with crucial elements of the apoptotic signaling pathway [44]. The findings also indicate that the potential biomarkers play significant roles in the development of SCM.

The authors recognize that this experiment is subject to some limitations. First, despite our strict modeling criteria, there is some heterogeneity in the LPS-induced disease outcome. Nevertheless, the high degree of homogeneity of the other samples may bolster the credibility of our results. Then, although the expression of hub genes during SCM has been probed, and their function preliminary explored, their functional pathways require further validation. However, numerous causal connections between biomarkers and disease are still awaiting validation. These will be the focus of future research.

## Conclusion

Genes associated with the pathogenesis of SCM were selected by a comprehensive analysis of transcriptomics and proteomics data. A total of 142 significant DEGs and 65 DEPs were identified. Through an integrated analysis of transcriptomic and proteomic data, we recognized six significantly differentially expressed targets at the mRNA and protein levels, which were mainly enriched in complement and coagulation cascades, and mainly associated with inflammation, stress responses, acute phase responses, and immune responses. The validity of these six genes was confirmed through RT-qPCR and WB. Furthermore, our results indicate that these genes including *Fga*, *Hspb1*, *C2*, *A2m*, *Hp* and *Orm1* may play crucial roles in the onset and progression of SCM. In conclusion, our research offers valuable insights into the pathogenesis of SCM and useful information for targeted prevention and treatment of this disease.

## Supporting information

S1 Fig PCA score plot of the transcriptome. Samples between Ctrl and LPS groups are plotted.
(PDF)

S1 Raw images Raw western blots. (PDF)

S1 Table Primers for qPCR. (XLS)

S2 Table The RPKM of genes analyzed in the heart of rats. (XLS)

S3 Table DE genes in the Ctrl and LPS groups in the heart. (XLS)

S4 Table The information of proteins identified in the heart of rats. (XLS)

S5 Table DE proteins in the Ctrl and LPS groups in the heart. (XLS)
