# Supplementary figures and images for: Integrative omics analysis identifies biomarkers of septic cardiomyopathy
Source: PLoS One. 2024 Nov 15;19(11):e0310412. doi: 10.1371/journal.pone.0310412 (PMC11567565; doi:10.1371/journal.pone.0310412)

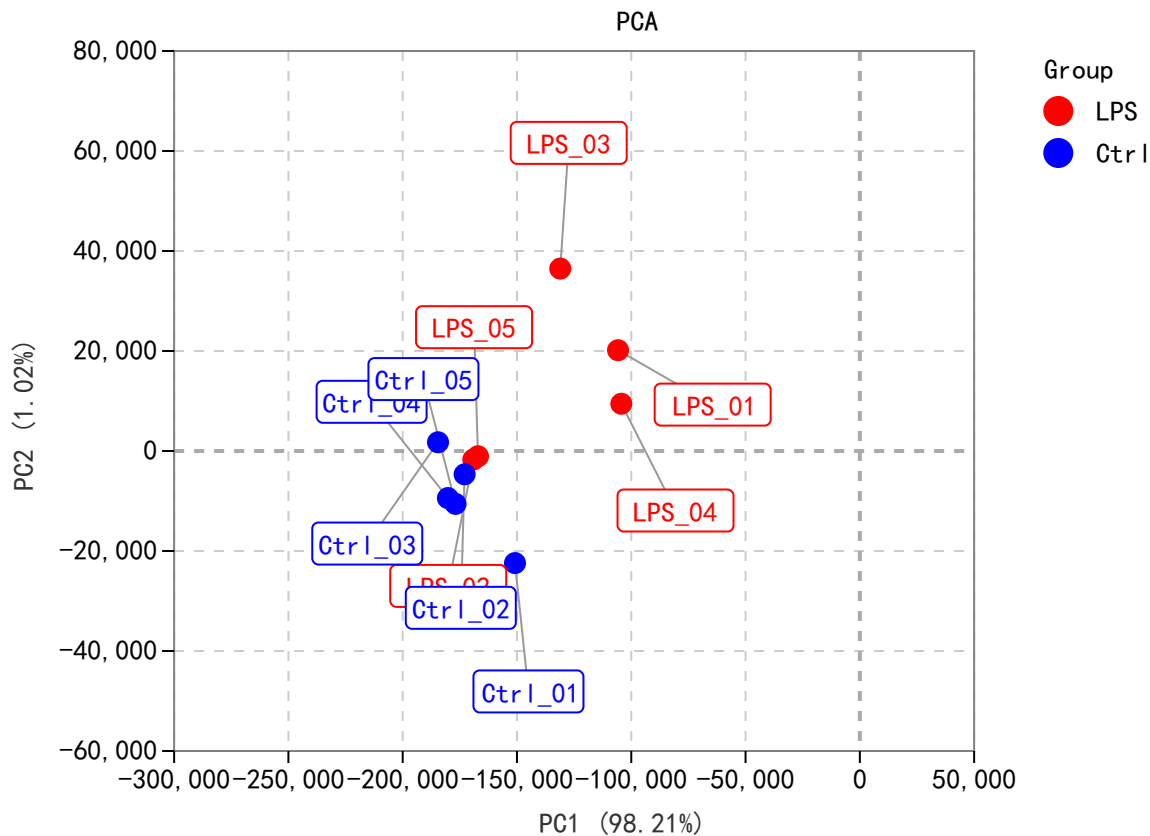

Supplement: S1 Fig — Samples between Ctrl and LPS groups are plotted. (PDF) [file pone.0310412.s001.pdf]
