# Supplementary material for: Integrative omics analysis identifies biomarkers of septic cardiomyopathy
Source: PLoS One. 2024 Nov 15;19(11):e0310412. doi: 10.1371/journal.pone.0310412 (PMC11567565; doi:10.1371/journal.pone.0310412)
Supplement: S1 Raw images — (PDF) [file pone.0310412.s002.pdf]

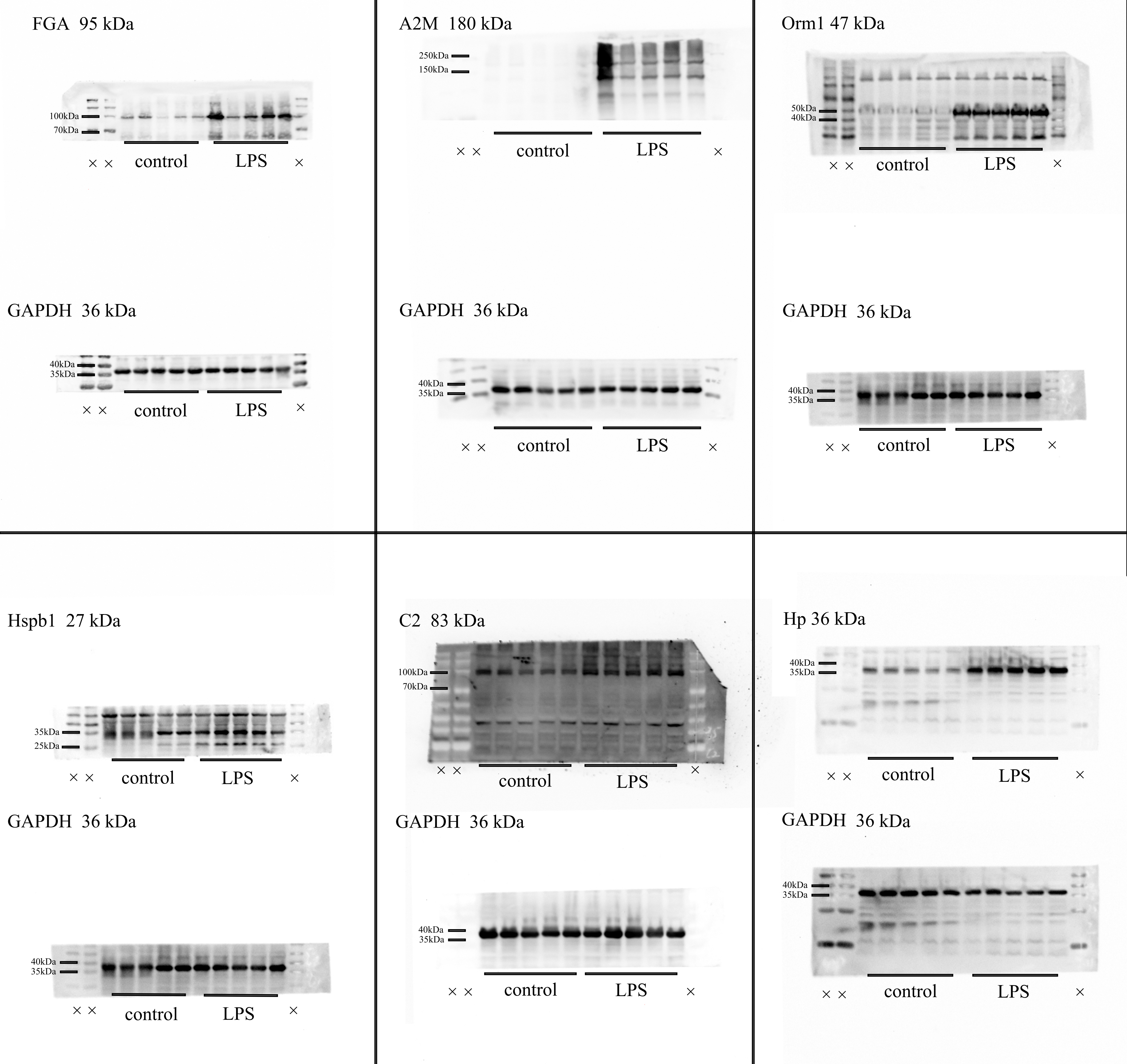

FGA 95 kDa
100kDa
70kDa
× × control LPS ×
GAPDH 36 kDa
40kDa
35kDa
× × control LPS ×
A2M 180 kDa
250kDa
150kDa
× × control LPS ×
GAPDH 36 kDa
40kDa
35kDa
× × control LPS ×
Orm1 47 kDa
50kDa
40kDa
× × control LPS ×
GAPDH 36 kDa
40kDa
35kDa
× × control LPS ×
Hspb1 27 kDa
35kDa
25kDa
× × control LPS ×
GAPDH 36 kDa
40kDa
35kDa
× × control LPS ×
C2 83 kDa
100kDa
70kDa
× × control LPS ×
GAPDH 36 kDa
40kDa
35kDa
× × control LPS ×
Hp 36 kDa
40kDa
35kDa
× × control LPS ×
GAPDH 36 kDa
40kDa
35kDa
× × control LPS ×
